# An *ACACB* Variant Implicated in Diabetic Nephropathy Associates with Body Mass Index and Gene Expression in Obese Subjects

**DOI:** 10.1371/journal.pone.0056193

**Published:** 2013-02-27

**Authors:** Lijun Ma, Mariana Murea, James A. Snipes, Alejandra Marinelarena, Jacqueline Krüger, Pamela J. Hicks, Kurt A. Langberg, Meredith A. Bostrom, Jessica N. Cooke, Daisuke Suzuki, Tetsuya Babazono, Takashi Uzu, Sydney C. W. Tang, Ashis K. Mondal, Neeraj K. Sharma, Sayuko Kobes, Peter A. Antinozzi, Matthew Davis, Swapan K. Das, Neda Rasouli, Philip A. Kern, Nathan J. Shores, Lawrence L. Rudel, Matthias Blüher, Michael Stumvoll, Donald W. Bowden, Shiro Maeda, John S. Parks, Peter Kovacs, Robert L. Hanson, Leslie J. Baier, Steven C. Elbein, Barry I. Freedman

**Affiliations:** 1 Wake Forest School of Medicine, Winston-Salem, North Carolina, United States of America; 2 PECRB, National Institute of Diabetes and Digestive and Kidney Diseases, Phoenix, Arizona, United States of America; 3 Interdisciplinary Center for Clinical Research, University of Leipzig, Leipzig, Germany; 4 Division of Nephrology and Metabolism, Department of Internal Medicine, Tokai University, Tokyo, Japan; 5 The Diabetes Center, Tokyo Women's Medical University, Tokyo, Japan; 6 Department of Medicine, Shiga University of Medical Science, Otsu, Shiga, Japan; 7 Department of Internal Medicine, University of Hong Kong Queen Mary Hospital, Hong Kong; 8 Department of Internal Medicine, University of Colorado School of Medicine, Aurora, Colorado, United States of America; 9 Department of Internal Medicine, University of Kentucky College of Medicine, Lexington, Kentucky, United States of America; 10 Transplant Hepatology, Abdominal Transplant Institute, Tulane Medical Center, New Orleans, Louisiana, United States of America; 11 Laboratory for Endocrinology and Metabolism, RIKEN Center for Genomic Medicine, Yokohama, Japan; Karolinska Institutet, Sweden

## Abstract

Acetyl coenzyme A carboxylase B gene (*ACACB*) single nucleotide polymorphism (SNP) rs2268388 is reproducibly associated with type 2 diabetes (T2DM)-associated nephropathy (DN). *ACACB* knock-out mice are also protected from obesity. This study assessed relationships between rs2268388, body mass index (BMI) and gene expression in multiple populations, with and without T2DM. Among subjects without T2DM, rs2268388 DN risk allele (T) associated with higher BMI in Pima Indian children (n = 2021; p-additive = 0.029) and African Americans (AAs) (n = 177; p-additive = 0.05), with a trend in European Americans (EAs) (n = 512; p-additive = 0.09), but not Germans (n = 858; p-additive = 0.765). Association with BMI was seen in a meta-analysis including all non-T2DM subjects (n = 3568; p-additive = 0.02). Among subjects with T2DM, rs2268388 was not associated with BMI in Japanese (n = 2912) or EAs (n = 1149); however, the T allele associated with higher BMI in the subset with BMI≥30 kg/m^2^ (n = 568 EAs; p-additive = 0.049, n = 196 Japanese; p-additive = 0.049). Association with BMI was strengthened in a T2DM meta-analysis that included an additional 756 AAs (p-additive = 0.080) and 48 Hong Kong Chinese (p-additive = 0.81) with BMI≥30 kg/m^2^ (n = 1575; p-additive = 0.0033). The effect of rs2268388 on gene expression revealed that the T risk allele associated with higher *ACACB* messenger levels in adipose tissue (41 EAs and 20 AAs with BMI>30 kg/m^2^; p-additive = 0.018) and ACACB protein levels in the liver tissue (mixed model p-additive = 0.03, in 25 EA bariatric surgery patients with BMI>30 kg/m^2^ for 75 exams). The T allele also associated with higher hepatic triglyceride levels. These data support a role for *ACACB* in obesity and potential roles for altered lipid metabolism in susceptibility to DN.

## Introduction

There is a world-wide epidemic of type 2 diabetes mellitus (T2DM) and chronic diabetes complications are major public health concerns [1]. Dyslipidemia associates with risk for T2DM and its complications, particularly diabetic nephropathy (DN) [2]. ACACB is the rate-limiting enzyme for fatty acid oxidation, and single nucleotide polymorphism (SNP) rs2268388 in the acetyl coenzyme A carboxylase B gene (*ACACB*) is reproducibly associated with T2DN and diabetic end-stage renal disease (ESRD) [3], [4]. Relative to the C allele, a 29-bp DNA fragment containing the T risk allele of rs2268388 demonstrated greater enhancer activity in cultured human renal proximal tubular epithelial cells, indicating higher ACACB expression in risk allele carriers [3]. Genome-wide association studies (GWAS) and the HapMap database have not revealed other SNPs in high genotypic concordance with rs2268388. In addition, the T allele of rs2268388 is overrepresented in obese women with T2DM and had higher transcription binding affinity than the C allele [5]. Therefore, rs2268388 could contribute to regulation of metabolism, alterations in lipids, and adiposity. We prioritized this SNP in order to explore potential links between predisposition to DN and lipid dysregulation in subjects with T2DM.

We previously examined association of rs2268388 and ten other common coding *ACACB* variants with metabolic traits and gene expression [6]. Although significant associations were detected with gene expression and several metabolic traits for coding variants, we failed to associate rs2268388 with expression quantitative trait loci (QTL) or metabolic traits. This may have been due to low allele frequencies with reduced power or undetected gene*environment interactions [6].

To assess the role of the DN-associated *ACACB* SNP rs2268388 on body mass index (BMI), we tested for association between this SNP and BMI in four population groups, including Europeans and European Americans (EAs), African Americans (AAs), Pima Indians and Asians. Subjects with and without T2DM were assessed, with a focus on those with high BMI. Effects on gene expression were investigated in multiple tissues and cell types.

## Materials and Methods

### Study samples

**Table 1** contains demographic data in study participants. All participants provided written informed consent.

**Table 1:** Demographic data of the study cohorts.

Cohort	Status	N(M/F)	BMI(kg/m^2^)	Age(yrs)
Leipzig German	Non-diabetic	858(272/586)	28.70±5.70	49.0±13.5
Arkansas EAs	Non-diabetic	404(129/275)	30.30±5.92	38.96±10.36
Utah EAs	Non-diabetic	108(42/66)	27.51±5.63	40.13±11.32
Arkansas AAs	Non-diabetic	177(71/106)	30.68±6.07	39.38±9.44
Pima Indians	Non-diabetic children	2021(891/1130)	27.01±6.31*	13.85±3.98**
Arkansas adipose biopsy sample	Non-diabetic (All EAs)	105(43/62)	27.78±5.59	40.13±10.91
	Non-diabetic (All AAs)	44(27/17)	30.20±6.38	43.32±9.14
	Non-diabetic (EAs BMI>30 kg/m^2^)	41(14/27)	34.50±3.79	42.30±9.60
	Non-diabetic (AAs BMI>30 kg/m^2^)	20(9/11)	35.82±4.37	43.70±9.39
Wake Forest liver biopsy sample EAs		26(20/6)	43.14±11.61	50.08±9.91
Wake Forest liver biopsy sample AAs		3(1/2)	43.36±8.82	45.00±10.54
Wake Forest EAs	Diabetic (All)	1149(555/594)	31.02±7.13	64.21±9.82
	Diabetic (BMI>30 kg/m^2^)	568(236/332)	36.57±5.60	61.65±8.76
Wake Forest AAs	Diabetic (All)	1446(593/853)	32.43±14.29	59.41±10.41
	Diabetic (BMI>30 kg/m^2^)	756(259/497)	38.56±17.10	57.27±9.85
Japanese	Diabetic (All)	2912(1697/1215)	24.0±3.8	63.2±10.7
	Diabetic (BMI>30 kg/m^2^)	196(82/114)	32.8±3.3	55.3±13.1
HK Chinese	Diabetic (All)	596(317/279)	24.56±4.20	66.82±10.90
	Diabetic (BMI>30 kg/m^2^)	48(29/19)	33.83±4.53	60.32±12.20
Pima Indians	Non-diabetic Pima children eventually developed DM	642(259/383)	max adult BMI 40.64±8.79***BMI at first DM visit 38.88±8.41	age at max adult BMI 33.67±9.97age at first DM visit 32.78±9.45
Pima Indians All		3197(1353/1844)	max BMI 37.02±8.75	age at max BMI 35.06±14.21

EA: European American; AA: African American.

* The analysis used age & sex standardized z scores (mean: 0.288±1.04). Mean shown is mean of max z-scores converted to BMI units using mean & SD of 12 year-old females.

** Age shown is age at max BMI z-score; not necessarily same as age at max BMI.

*** max BMI from age >15 yrs. N = 637 since 5 kids had no exams after age 15.

### Wake Forest School of Medicine (WFSM) Diabetes Heart Study (DHS) and African American-Diabetes Heart Study (AA-DHS)

Subjects with T2DM in the DHS (n = 1149 EAs), AA-DHS and AA T2D-ESRD studies (n = 1446 AAs) were born in North Carolina, South Carolina, Georgia, Tennessee, or Virginia. Those with a history of ketoacidosis or developing DM before the age of 25 years and receiving continuous insulin treatment since diagnosis were excluded. For patients with ESRD, maximal pre-dialysis BMI values were utilized. Study procedures were approved by the WFSM Institutional Review Board (IRB) [7], [8].

### University of Utah (UT) and University of Arkansas (AR) samples

EAs (n = 512) and AAs (n = 177) without T2DM ascertained in UT and AR were evaluated under separate IRB-approved protocols at the University of Utah Health Sciences Center and University of Arkansas for Medical Sciences (UMAS) [9]. Participants had normal 75 g oral glucose tolerance tests (OGTTs). A subset of the AR sample underwent adipose biopsy (below).

### Pima Indian population-based samples

A population-based sample of full-heritage Pima Indians (n = 3,197) derived from the longitudinal study of the etiology of T2DM in the Gila River Indian Community in Central Arizona was evaluated [10]. This study was approved by the National Institute of Diabetes and Digestive and Kidney Diseases IRB and included related individuals with biennial exams measuring height, weight, and a 75-g OGTT (WHO 1999 criteria). In the sample of full-heritage Pima Indians, BMI was included for exams after the age of 5 years (19,385 BMI measures in 3,197 subjects). In a subset of the full sample, Pima children aged 5–19 years had their maximum BMI recorded at a time when they were not yet diagnosed with T2DM (n = 2,021).

### German samples

Non-diabetic Europeans (n = 858) recruited at University Hospital in Leipzig, Germany were included in a study approved by the ethics committee. Diabetes was defined using WHO 1999 criteria.

### Japanese samples

Samples from outpatient diabetes clinics at Shiga University of Medical Science, Tokyo Women's Medical University, Kawai Clinic, and Tokai University Hospital were evaluated under IRB approved protocols [3]. Diabetes was defined using WHO 1999 criteria, and type 2 diabetes patients with normoalbuminuria or with microalbuminuria were included in the present analysis.

### Hong Kong samples

Chinese subjects born in Hong Kong or southern China were evaluated [4]. T2DM was diagnosed in those treated with oral hypoglycemic agents and/or insulin, in the absence of insulin-only treatment after one year of T2DM. The study was approved by the IRBs at University of Hong Kong/Hospital Authority Hong Kong West, Kowloon Central and East Clusters of Hospitals and met criteria in the Declaration of Helsinki.

### Genotyping

SNP rs2268388 was genotyped in WFSM and Hong Kong samples using the MassARRAY genotyping system (Sequenom Inc., San Diego, CA). PCR primers were designed using the MassARRAY Assay Design 3.4 Software (Sequenom Inc., San Diego, CA). The minimum SNP call rate for an individual was 98.4%. Forty-six blind duplicates were genotyped with a concordance rate of 99.6%. The SNP was genotyped in UT, AR, Pima Indian and German samples using the TaqMan genotyping reaction. DNA was amplified on a GeneAmp PCR system 9700 (95°C for 10 min, followed by 40 cycles of 95°C for 30 s and 60°C for 1 min 30 s), and fluorescence was detected on an ABI Prism 7700 or 7500 (Applied BioSystems). Sequence information for oligonucleotide primers and probes is available upon request. The overall genotyping call rate exceeded 98%. Duplicate quality control (QC) samples were randomly distributed in the UT/AR (n = 69) and Pima (n = 150) plates assuring ∼99% reproducibility. To assess genotyping reproducibility in the German cohort, a random ∼5% selection of the sample was re-genotyped and the genotypic concordance reached 100%. In the Japanese cohort, the SNP was genotyped using a multiplex-PCR-invader assay. The call rate for the SNP was 99.1% and a concordance rate in duplicate samples (n = 1,289) was 99.6% [3].

### Adipose Biopsy

149 AR non-diabetic subjects underwent a fasting adipose biopsy under local lidocaine anesthesia [11]. Tissue was obtained using a Bergstrom needle from abdominal sub-cutaneous fat. Samples were rinsed in sterile saline, quick frozen in liquid nitrogen and stored at −80°C.

### Liver Biopsy

Twenty-five EAs undergoing laparoscopic gastric banding or Roux-en-Y gastric bypass surgery at WFSM agreed to an intra-operative wedge liver biopsy in an IRB-approved study. Patients with liver disease other than non-alcoholic fatty liver disease (macrosteatosis in the setting of hepatic ballooning, inflammation or fibrosis), malignancy, coagulopathy or need for anticoagulation, chronic inflammatory diseases, or ethanol intake ≥105 g/week (or ≥45 g/day) were excluded. Hepatic lipid analysis methods in these wedge biopsies immediately snap frozen and stored at −80°C have been reported [12]. Briefly, lipids from 50–100 mg of minced tissue were extracted in 2∶1 chloroform∶methanol overnight and triglycerides (TGs) quantified using enzymatic assays after addition of Triton-X100 (TG and cholesterol - Roche Diagnostics; unesterified cholesterol - Wako Chemicals USA) [13], [14]. TG content was calculated as mg (TG)/g liver total cell lysate protein.

### RNA isolation and gene transcription arrays

Total RNA was isolated from adipose using the RNAeasy Lipid Tissue Mini kit (Qiagen, Valencia, CA). The quantity and quality of the isolated RNA were determined by ultraviolet spectrophotometry and electrophoresis using an Agilent 2100 Bioanalyzer (Agilent Technologies, Santa Clara, CA). Genome-wide transcriptome analysis was performed on the AR adipose biopsy sample using Human HT-12 v4 Expression BeadChip (Illumina, San Diego,CA) whole-genome gene expression array according to the vendor-recommended protocol.

### Western Blot

Liver samples were homogenized in 5× volume of phosphate-buffered saline relative to tissue weight and protease inhibitors added. Sodium dodecyl sulfate buffer (1% sodium dodecyl sulfate, 10 mmol/L Tris, pH 7.6) was added in the amount of the homogenate and sonicated. Equal amounts of protein for each sample were separated by 4% to 20% sodium dodecyl sulfate-polyacrylamide (SDS) gel electrophoresis, transferred onto a nitrocellulose membrane (Bio-Rad, Hercules, CA) and blocked for 1 hour at room temperature with Tris-buffered saline containing 1% skim milk powder, 0.1% Tween 20. Blots were incubated overnight at 4°C with a polyclonal anti-ACACB antibody (1∶1,000, Sigma, HPA006554). Membranes were washed 3 times in Tris-buffered saline containing 0.1% Tween 20 and incubated for 1 hour in the blocking buffer with anti-rabbit IgG conjugated to horseradish peroxidase (1∶20,000; Jackson Immuno-Research, West Grove, PA, USA). The bound antibodies were visualized using enhanced chemiluminescence (Super Signal West Pico; Thermo Pierce, Rockford, IL, USA) and recorded on X-ray film. The bands were scanned and densities quantitated using Image J (http://rsbweb.nih.gov/ij/). Liver lysate samples were randomized before being loaded on the SDS gels for three batches of replicated experiments.

### Statistical Analyses

Statistical analyses were performed using SAS 9.1 software (SAS Institute; Cary, NC). A generalized linear model (GENMOD) was used to assess associations between genotype and BMI, *ACACB* gene expression in adipose and ACACB protein level in liver. For additive model analyses, homozygotes for allele (1/1), heterozygotes (1/2), and homozygotes for allele (2/2) were coded to a continuous variable (0, 1, and 2). Generalized estimating equations (GEE) were used to account for sibships in the UT EAs, Wake Forest, and Pima subjects, in addition to adjustment for age, sex, and BMI. The SNP was analyzed using the same genetic model in all analyses, regardless of the study sample. A meta-analysis was performed using the Stouffer method [15]. Mixed model analyses were applied for multiple exams of continuous variables (BMI for Pima Indians and ACACB protein levels by Western blot) to account for repeat values. P-values<0.05 were considered to represent nominal levels of statistical significance.

## Results

### Association with BMI in subjects without diabetes

**Figure 1:**
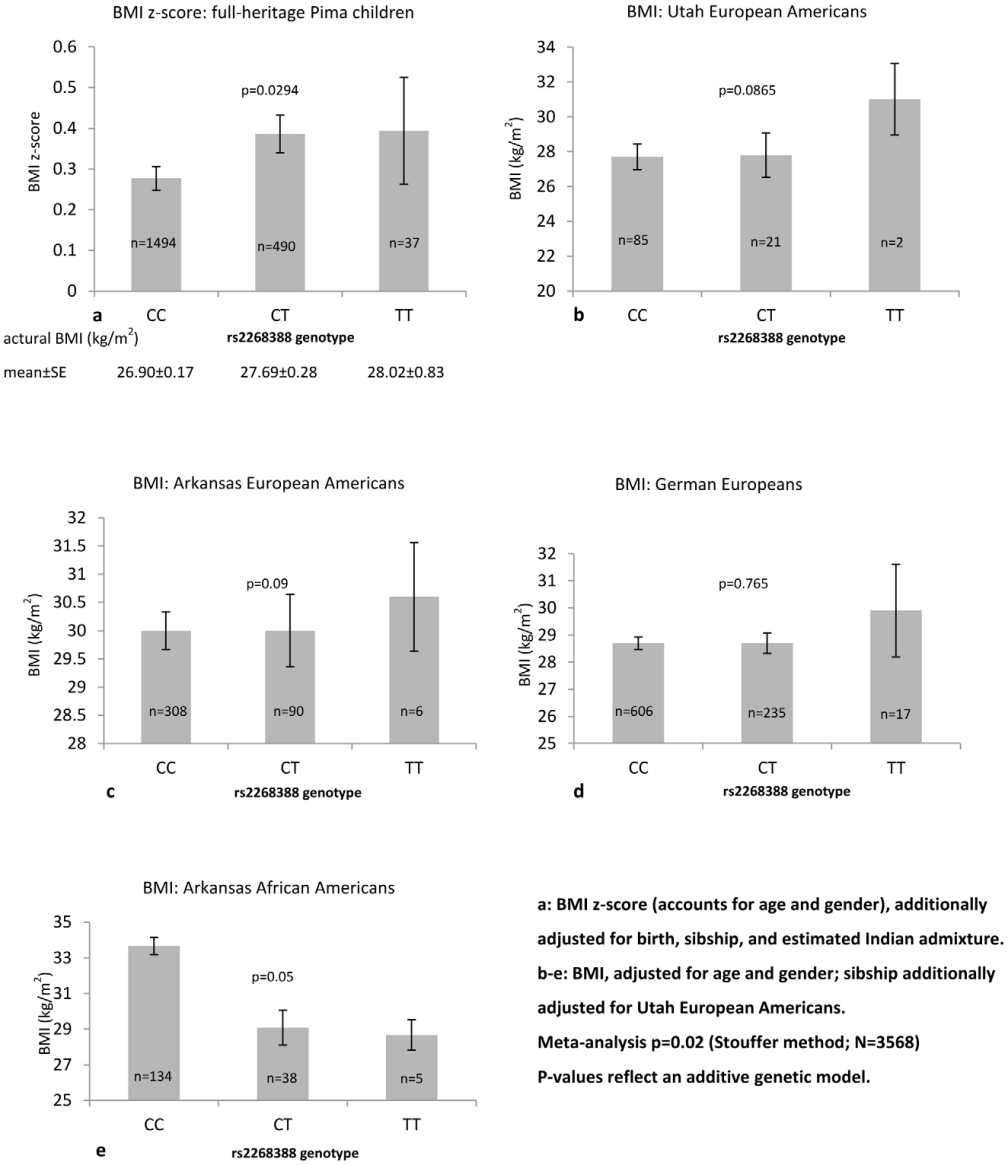
Association of rs2268388 with BMI in non-diabetic subjects.

**Figure 1** summarizes association results for BMI with rs2268388 in non-diabetic Germans (European), UT EAs, AR EAs, AR AAs and Pima Indians. Significant association was seen in Pima Indian children (p = 0.0294); while the three European-derived populations revealed non-statistically significant trends consistent in direction. Association in non-diabetic AR AAs was nominally significant but opposite in direction. A meta-analysis revealed significant association (p = 0.02 Stouffer method) combining all populations, mainly driven by Pima Indian children (**Figure 1**).

### Association with BMI in subjects with diabetes and BMI>30 kg/m^2^

**Figure 2:**
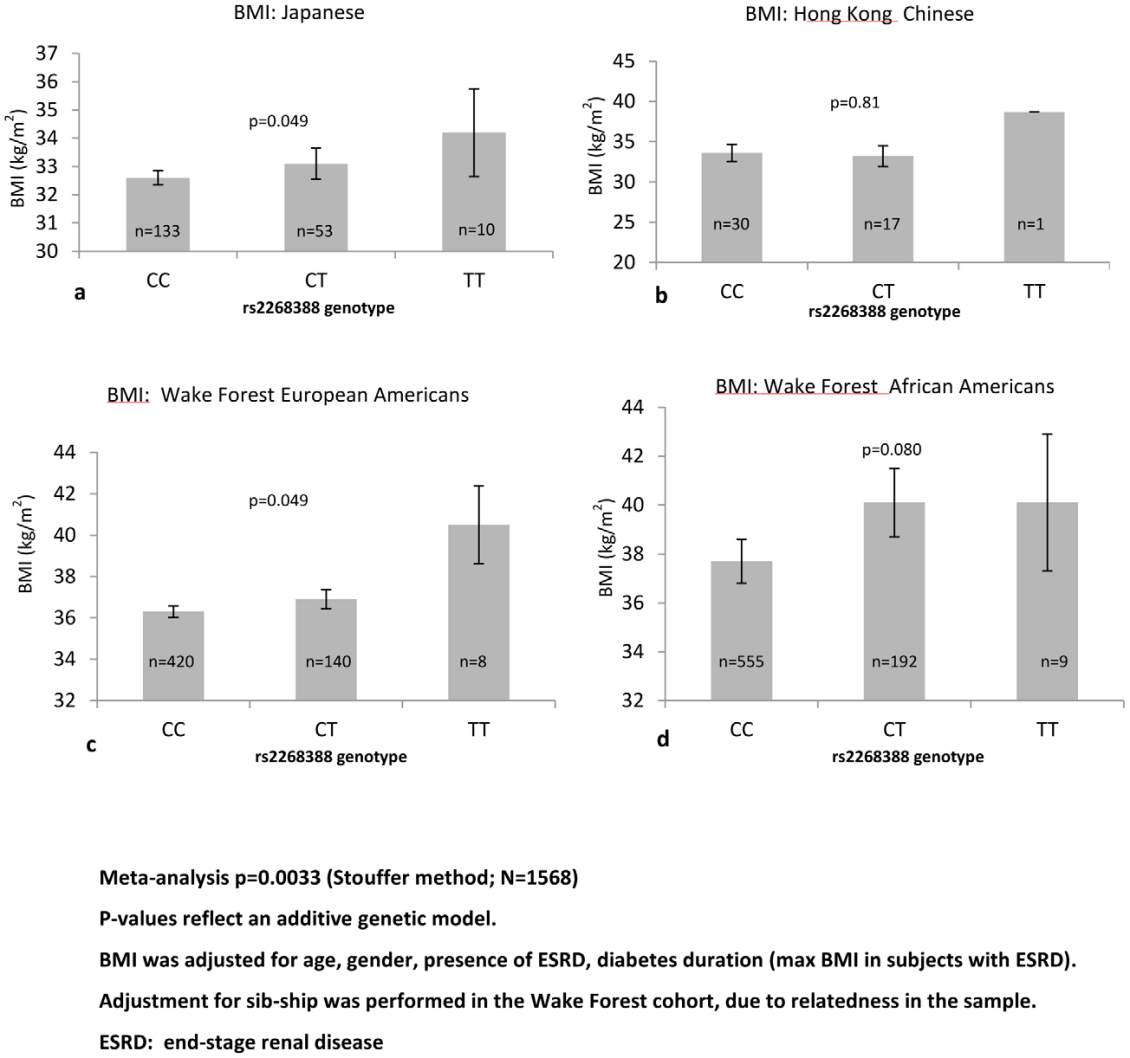
Association of rs2268388 with BMI in subjects with type 2 diabetes and BMI≥30 kg/m^2^.

No significant association of BMI with rs2268388 was detected in diabetic subjects within each cohort or the combined sample (data not shown). However, stratified analyses revealed rs2268388 associated with BMI in subjects with T2DM and BMI≥30 kg/m^2^ in Japanese (p = 0.049) and WFSM EAs (p = 0.049), with a non-significant trend in WFSM diabetic AAs (p = 0.08). No association was seen in Hong Kong Chinese (p = 0.81), but the direction was consistent. A meta-analysis in combined WFSM AAs, WF EAs, Japanese and Hong Kong Chinese with T2DM and BMI≥30 kg/m^2^ revealed significant evidence of association (p = 0.0033; **Figure 2**).

### Association with BMI in Pima Indians

BMI z-scores were evaluated in all full-heritage Pima subjects <18 years of age who had at least one non-diabetic exam and who then subsequently developed T2DM (Model 1, n of subjects = 642, N of exams = 5321). In addition, all BMI exams in all recorded full-heritage Pima Indians genotyped for rs2268388 were evaluated in Model 2 (n of subjects = 3197, N of exams = 19385). BMI was not associated with rs2268388 in Model 1 (mixed model analysis p = 0.265, **Table 2**). However, when all BMI exam z-scores were examined in Model 2, a trend toward association was seen (mixed model analysis p = 0.0755; **Table 2**). Subjects in model 1 had diabetes, with BMIs recorded longitudinally after their initial non-diabetic exams. Pima Indians have a high prevalence of T2D; a majority will ultimately develop T2DM. Therefore, we included both models with the full WF AA, WF EA, Japanese and Hong Kong Chinese T2DM study samples. A meta-analysis for BMI and rs2268388 in all subjects with T2DM revealed significant association (Stouffer method p = 0.0022 with Model 1 and p = 0.01 with Model 2, respectively; **Table 2**).

**Table 2:** Association of rs2268388(C/T) with BMI in full-heritage Pima Indian longitudinal cohort.

Analysis	CC	CT	TT	Beta	P-value
	BMI z-score±SE	BMI z-score ±SE	BMI z-score ±SE		
	(n_CC/N_exams)	(n_CT/N_exams)	(n_TT/N_exams)		
model 1	0.253±0.038	0.373±0.058	0.047±0.206	0.065	0.26531
	(470/3987)	(162/1257)	(10/77)		
Meta-analysis together with 4 cohorts (Japanese, HK Chinese, Wake Forest EAs and AAs) in Figure 2 (Stouffer method)					0.0022
model 2	−0.002±0.020	0.063±0.031	0.039±0.097	0.052	0.07551
	(2367/14422)	(765/4565)	(65/398)		
Meta-analysis together with 4 cohorts (Japanese, HK Chinese, Wake Forest EAs and AAS) in Figure 2 (Stouffer method)					0.01

model 1: > = 1 nondiabetic exam age <18 and subsequently developed DM; total n = 642; total exam N = 5321;

P values were adjusted for age, gender, sibship, and repeated exams (matrix: autoregressive; additive genetic model).

model 2: All individuals- all examinations; total n = 3197; total exam N = 19385;

P values were adjusted for age, gender, sibship, diabetic status, duration of diabetes, and repeated exams (matrix: autoregressive; additive genetic model).

T allele is defined as risk allele for higher BMI z-score.

n_CC, n_CT, n_TT: number of individuals in each genotypic group. N_exams: number of exams.

### Association with *ACACB* messenger level in non-diabetic subjects

**Figure 3:**
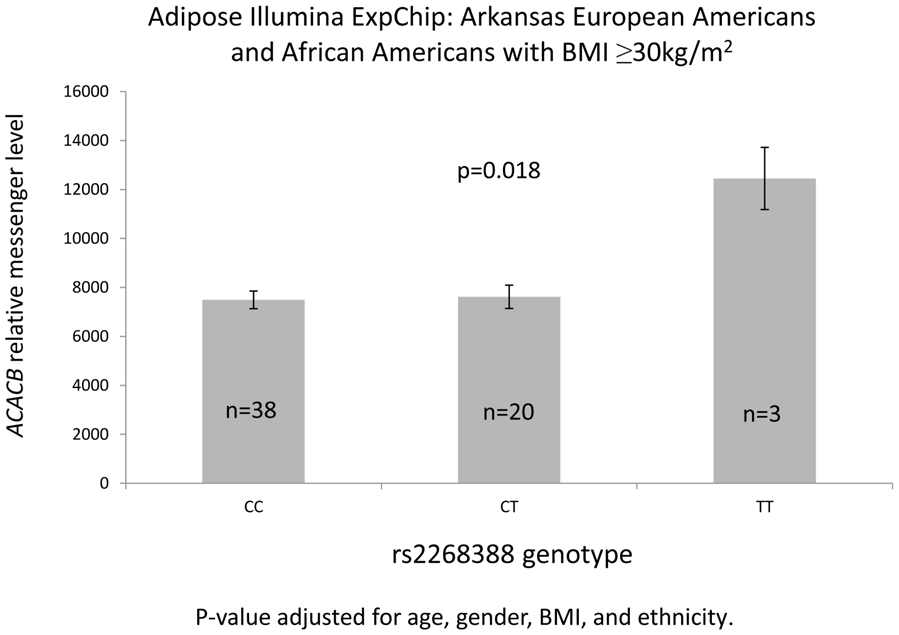
Adipose ACACB expression by rs2268388.

The messenger level of *ACACB* was measured by Human HT-12 v4 BeadChip (Illumina, San Diego,CA) whole-genome gene expression array in AR non-diabetic EAs and AAs with subcutaneous adipose tissue (all had an OGTT). Significant association was not observed in the full biopsy sample (n = 149, data not shown). A nominally significant association between *ACACB* messenger level and rs2268388 was seen in subjects with BMI≥30 kg/m^2^ (p = 0.018) adjusting for age, gender, BMI, and ethnicity (**Figure 3**), where the (T) risk allele for higher *ACACB* messenger level was consistent with associations with higher BMI.

### Association with liver ACACB protein level

**Figure 4:**
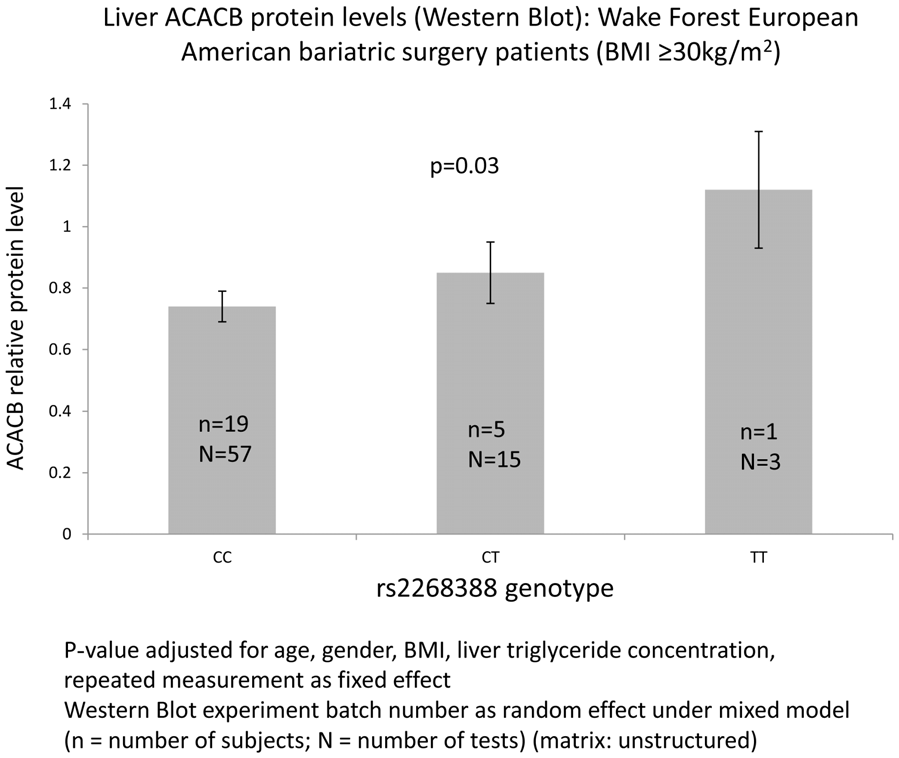
Liver ACACB expression by rs2268388.

**Figure 4** reveals significant association between liver ACACB protein level and rs2268388 in EAs who underwent bariatric surgery (BMI>30 kg/m^2^, n = 25 with 75 total Western blot exams; p = 0.03). The (T) risk allele for higher ACACB protein level was consistent with associations with higher BMI.

### Association with hepatic triglycerides (TGs)

**Figure 5:**
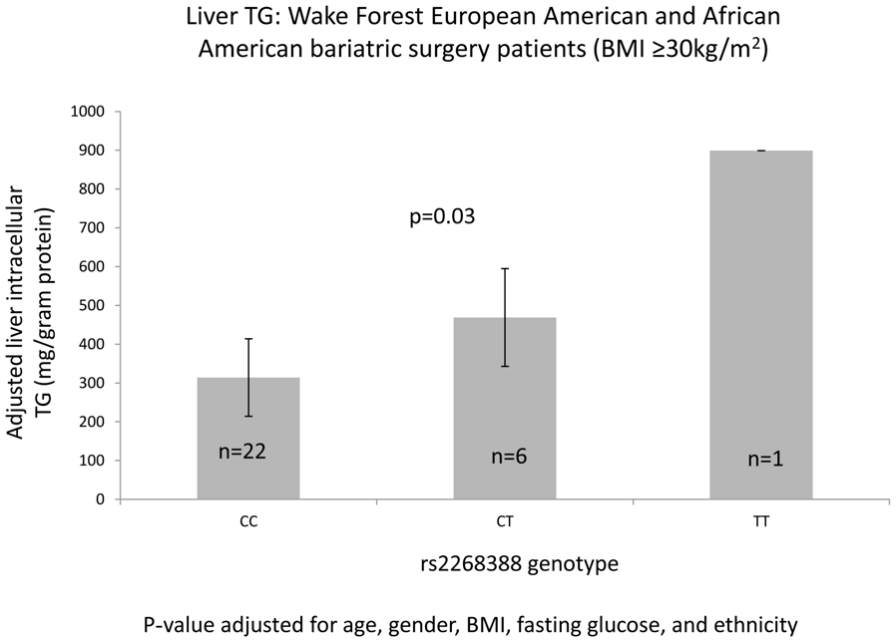
Liver cell triglyceride (TG) by rs2268388.

**Figure 5** reveals that rs2268388 was associated with higher hepatic intracellular TGs in 26 EA and 3 AA subjects who underwent bariatric surgery (BMI>30 kg/m^2^; p = 0.03). The (T) risk allele for higher TG was consistent with associations with higher BMI.

## Discussion

ACACB is a rate-limiting enzyme involved in mitochondrial fatty acid oxidation and plays a key role in fatty acid metabolism [16]–[19]. The current results reveal that *ACACB* SNP rs2268388 (reproducibly implicated in risk of nephropathy) associates with BMI in general populations and with obesity in subjects with T2D, as well as impacts gene expression in adipose and hepatic tissue. *ACACB cis* SNPs reportedly regulate *ACACB* gene expression, providing potential roles for effects on adiposity [6]. Adipose *ACACB* expression was negatively associated with adiposity (p = 0.0002, r = −0.35) in EAs [6] and Pima Indians (p = 4.3×10^−12^, r = −0.45, personal communication [20]). Feed-back regulation has been observed by significant down-regulation of *ACACB* mRNA with free fatty-acid (palmitate or oleate) treatment [21], as well as reductions in ACACB protein expression (data not shown). This implicates a complex cross-regulation between *ACACB* gene expression and adiposity.

Although *ACACB* is a physiological candidate gene for obesity and diabetes, significant association has not been observed in GWAS. This may reflect the effect of gene*gene or gene*environment interactions. A recent gene*environment interaction study of *ACACB* variants in metabolism suggests that undiscovered causative variants may have been overlooked [22]. Associations between *ACACB* and obesity in general populations can be masked by environmental factors such as lifestyle and eating behaviors linked with development of adiposity. *Acacb* knock-out mice are less likely to gain weight when fed high-fat diets compared to their wild type littermates; however, significant differences in body weight were not observed when fed normal chow [16]. This phenomenon has been observed in other rodent models where overweight phenotypes are conditional on diet [23], [24].

*ACACB* variant rs2268388 is reproducibly associated with DN [3], [4]. There is also evidence implicating altered lipid metabolism in the pathogenesis of DN [25], [26]. PPARA agonists (fibrates), up-regulating fatty acid oxidation, may improve DN in humans [27], [28] and animal models [29]. Accelerated DN was observed in mice lacking the peroxisome proliferator-activated receptor alpha [30]. The mechanism of *ACACB* association with DN remains unknown.

*ACACB* variant rs2268388 was also associated with severe obesity in Spanish women, no data were provided in men [5]. We chose to perform the association analyses for this SNP with BMI in multiple samples, including general populations of non-diabetic subjects, subjects with T2DM, and subjects with T2DM and BMI≥30 kg/m^2^. We selected non-diabetic Pima childhood max BMI z-score to represent non-diabetic BMI measures for Pimas, since adult BMIs are usually high and largely impacted by environmental factors, and are very close to the first visit diabetic BMIs. However, it is informative to know that significant association was not observed between rs2268388 and maximum BMI in adult Pima Indians when non-diabetic (detailed data not shown). Our finding of association with BMI in obese African American subjects with T2D was consistent with the report in Spanish women [5]. Women, not men, contributed to the significant association in AAs (**Figure S1-1**); whereas gender interaction was not observed in EAs (**Figure S1-2**). In full-heritage Pima subjects <18 years of age who had at least one non-diabetic exam and who then subsequently developed T2DM (Model 1), there was a weak trend that men, not women, with T allele were heavier, and genotype*gender interaction was borderline significant; however no significant gender interaction was observed in all full-heritage Pimas for all recorded BMI exams (Model 2) (**Table S1**). Due to limited sample power for obese diabetic subjects of the Japanese and Chinese cohorts, additional gene*gender interaction analysis was not attempted for those two ethnic groups. Severe obesity in the Spanish population was defined as BMI≥35 kg/m^2^, whereas we chose BMI≥30 kg/m^2^ to capture obese Japanese and Hong Kong Chinese subjects. Although association with high BMI in any single population was relatively weak, overall effects in combined samples appeared stronger. BMI data in Pima Indians from an on-going half-century long longitudinal study were included. Full-heritage Pima Indians have minimum admixture from other populations and many had BMI records starting in childhood. This allowed us to perform mixed model analyses testing association of rs2268388 with BMI z-scores from multiple exams in every individual, rather than a single exam in each subject. This was important since BMI changes during life. The majority of Pima subjects' BMI exceed 34 kg/m^2^ in adulthood for the younger generations (**Figure S2**) and 80% of those aged above 55 years have T2DM [31].

*ACACB* gene expression by genotype revealed the same pattern with BMI in our combined Arkansas EA and AA samples, the rs2268388 DN “T” risk allele represented higher expression levels with BMI≥30 kg/m^2^. Higher *ACACB* expression is linked to obesity and insulin resistance in a mouse model fed a high-fat diet [17]. *ACACB* RNA expression levels were also elevated in transformed lymphoblast cell lines from subjects with the T allele in a combined analysis of CEU (Utah European ancestry population), Japanese and Yoruba samples (HapMap-Sanger gene expression database, **Figure S3**) (http://www.hapmap.org & ftp://ftp.sanger.ac.uk/pub/genevar/). Two groups reported that *Acacb* knock-out mice had similar body weights as wild type mice [32], [33]. Molecular explanations for the phenotypic differences observed between Olson's model [32] of *Acacb* deletion and that of Abu-Elheiga et al [16] resistant to obesity, diabetes and insulin resistance are unclear. The *Acacb* biotin-binding site was deleted in both models, but using different approaches. The targeting strategy employed in the original study replaced only the exon containing the biotin binding motif [16]. RNA splicing across the targeting cassette could have left the mRNA in frame, resulting in a mutated but otherwise intact protein lacking a catalytic domain. Such a protein could have “dominant negative” activity toward ACACA [33] where both ACACB and ACACA activities are inhibited. This is consistent with the effects of soraphen, an inhibitor of both ACACA and ACACB which improved peripheral insulin sensitivity in mice fed a high-fat diet [34].

Our report benefits from a liver tissue repository allowing performance of a Western blot analysis assessing ACACB protein levels in hepatic cell lysates from obese subjects undergoing bariatric surgery (**Figure S4**). Subjects with the T risk allele tended to have higher ACACB protein levels in liver cells, consistent with association of this SNP with *ACACB* RNA levels in adipose tissue from non-diabetic EA and AA subjects with BMI≥30 kg/m^2^. In addition, bariatric surgery subjects with the T allele tended to have higher liver TG levels. Abu-Elheiga et al. reported that Acacb knock-out mice were also protected against fatty liver during high-fat, high-carbohydrate diet and *de novo* lipogenic conditions [35]. These data support that metabolic changes with mutant *ACACB* may be conditional on nutritional status. The *ACACB* pathway may be a useful target for ameliorating metabolic syndrome.

Limitations of this study were small numbers of Asian samples and all except the Pima lacked longitudinal BMI data. The sample numbers in our gene expression studies had limited statistical power. However, we estimate the liver cohort had approximately 0.80 genetic power for detecting 20% of the variation in protein expression levels (assuming type 1 error rate = 0.005) using mixed models for triplicate Western blot analyses under unstructured matrix and adjustment to account for across experiment variance.

We conclude that the T allele for *ACACB* SNP rs2268388 associates with higher BMI in subjects with diabetes and BMI≥30 kg/m^2^ and in general non-diabetic populations. The T allele also associates with higher *ACACB* expression in subjects with elevated BMI at the RNA (adipose) and protein levels (liver). Intracellular hepatic TGs are also elevated with this allele. GWAS for BMI, obesity and T2DM may not identify metabolism-related genes involved in these processes when nutritional status and lifestyles are assumed to be homogenous. *ACACB* variants may regulate gene expression and impact BMI; however, adiposity can also impact *ACACB* gene expression and balance the effects of genetic variation [5]. The *ACACB* rs2268388 association with DN has been replicated in multiple populations and the T risk allele for DN appears consistent with that of higher BMI, elevated liver TGs and metabolic cell gene expression. Alterations in lipid metabolism may jointly impact development of DN and obesity.

## Supporting Information

Figure S1 BMI vs. rs2268388 in Wake Forest diabetic men and women. S1-1. BMI vs. rs2268388 in Wake Forest diabetic African Americans (AAs) (a: men BMI>30 kg/m^2^; b: women BMI>30 kg/m^2^; c: men; d: women). S1-2. BMI vs. rs2268388 in Wake Forest diabetic European Americans (EAs) (BMI>30 kg/m^2^) (a: men; b: women).
(JPG)

Figure S2 BMI distribution by age for different birth era groups in non-diabetic full-heritage Pima Indians.
(JPG)

Figure S3 *ACACB* expression in transformed lymphoblast cell lines in HapMap/Sanger gene expression data set (in CEU, JPT, &YRI).
(JPG)

Figure S4 Liver ACACB protein level (an example gel image for Western Blot) in Caucasians (underwent bariatric surgery: BMI>30 kg/m^2^).
(JPG)

Table S1 Association of rs2268388(C/T) with BMI in full-heritage Pima Indian men and women.
(PDF)
