# Supplementary figures and images for: An ACACB Variant Implicated in Diabetic Nephropathy Associates with Body Mass Index and Gene Expression in Obese Subjects
Source: PLoS One. 2013 Feb 27;8(2):e56193. doi: 10.1371/journal.pone.0056193 (PMC3584087; doi:10.1371/journal.pone.0056193)

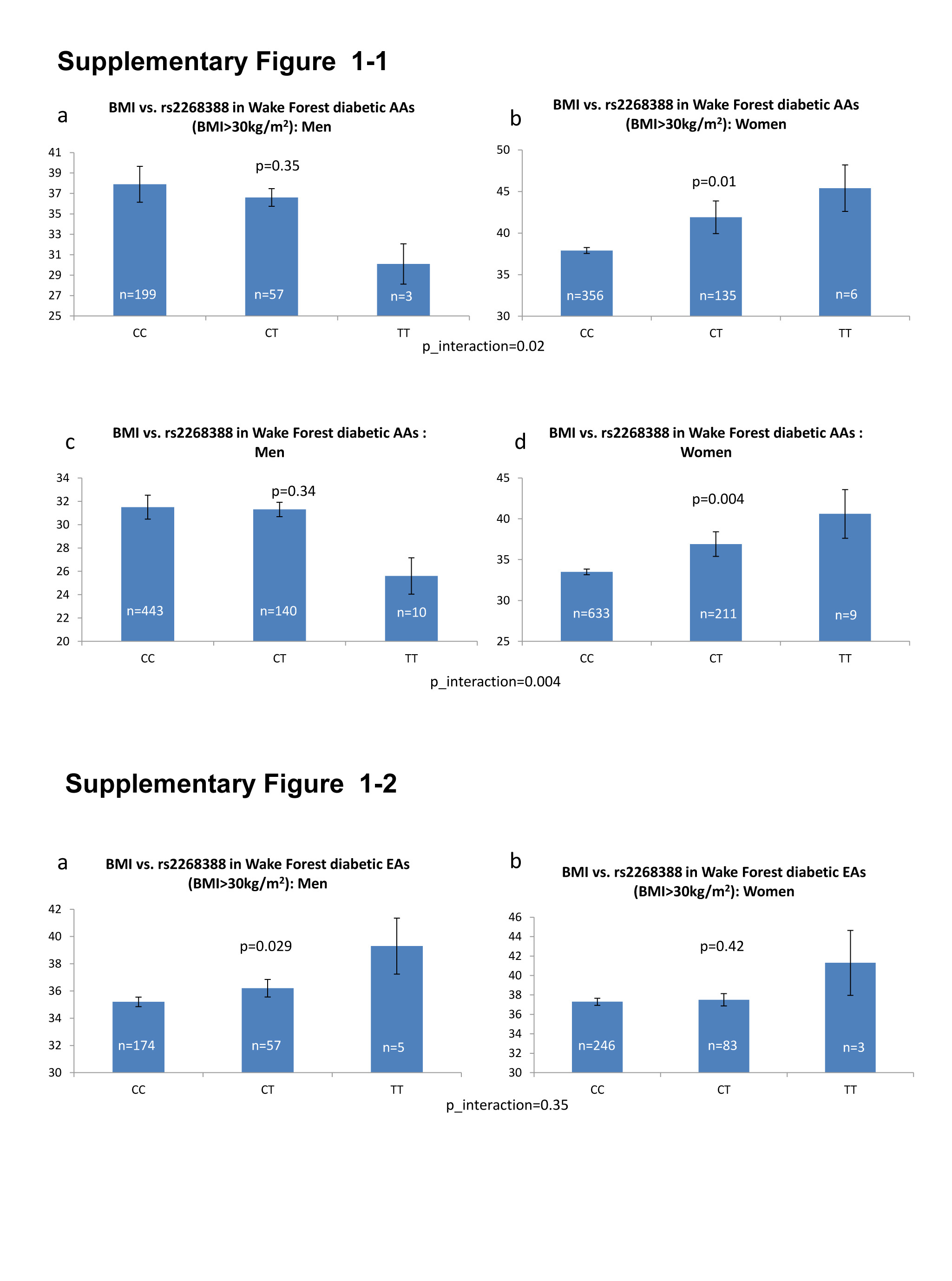

Supplement: Figure S1 — BMI vs. rs2268388 in Wake Forest diabetic men and women. S1-1. BMI vs. rs2268388 in Wake Forest diabetic African Americans (AAs) (a: men BMI>30 kg/m2; b: women BMI>30 kg/m2; c: men; d: women). S1-2. BMI vs. rs2268388 in Wake Forest diabetic European Americans (EAs) (BMI>30 kg/m2) (a: men; b: women). (JPG) [file pone.0056193.s001.jpg]

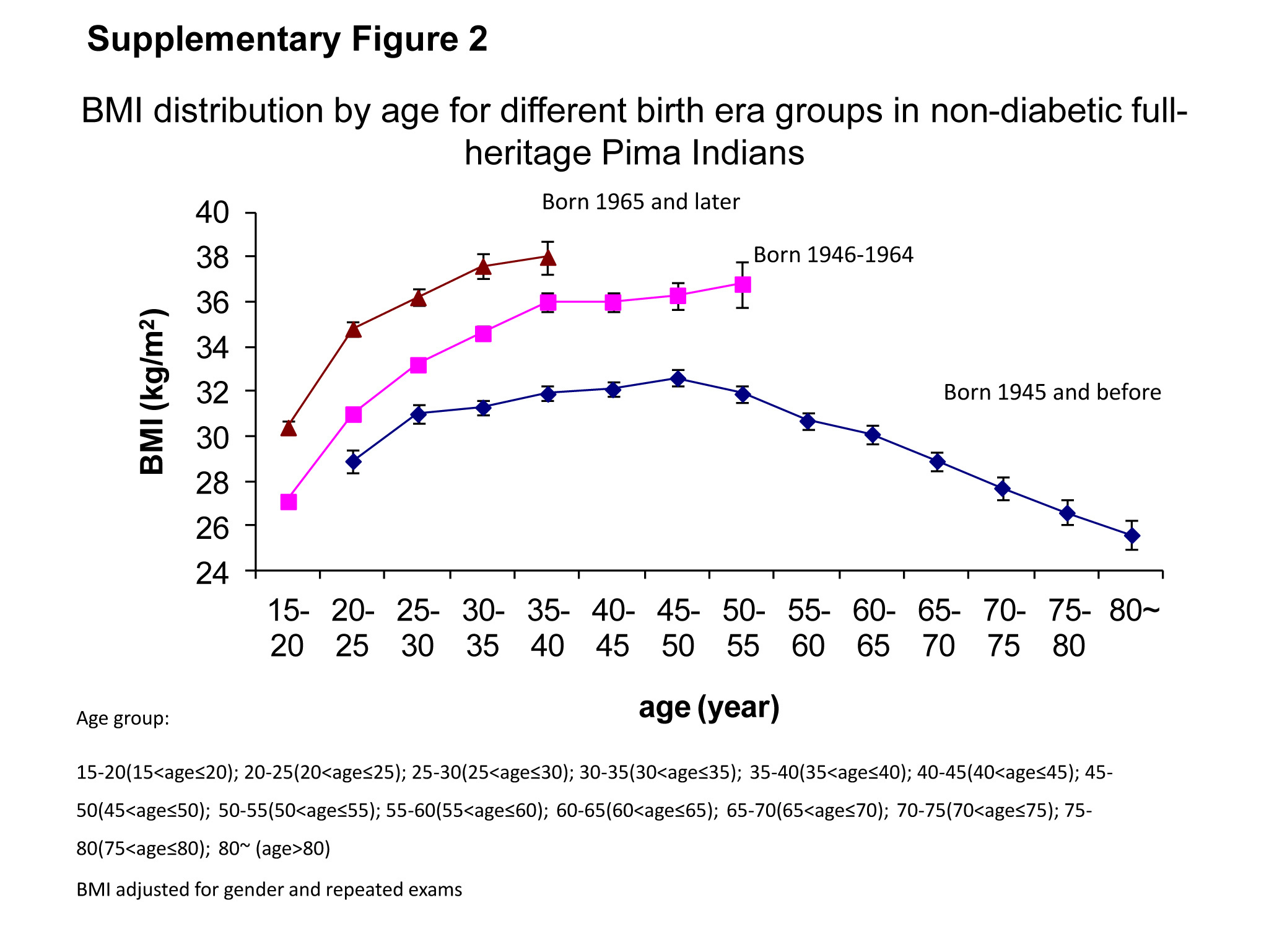

Supplement: Figure S2 — BMI distribution by age for different birth era groups in non-diabetic full-heritage Pima Indians. (JPG) [file pone.0056193.s002.jpg]

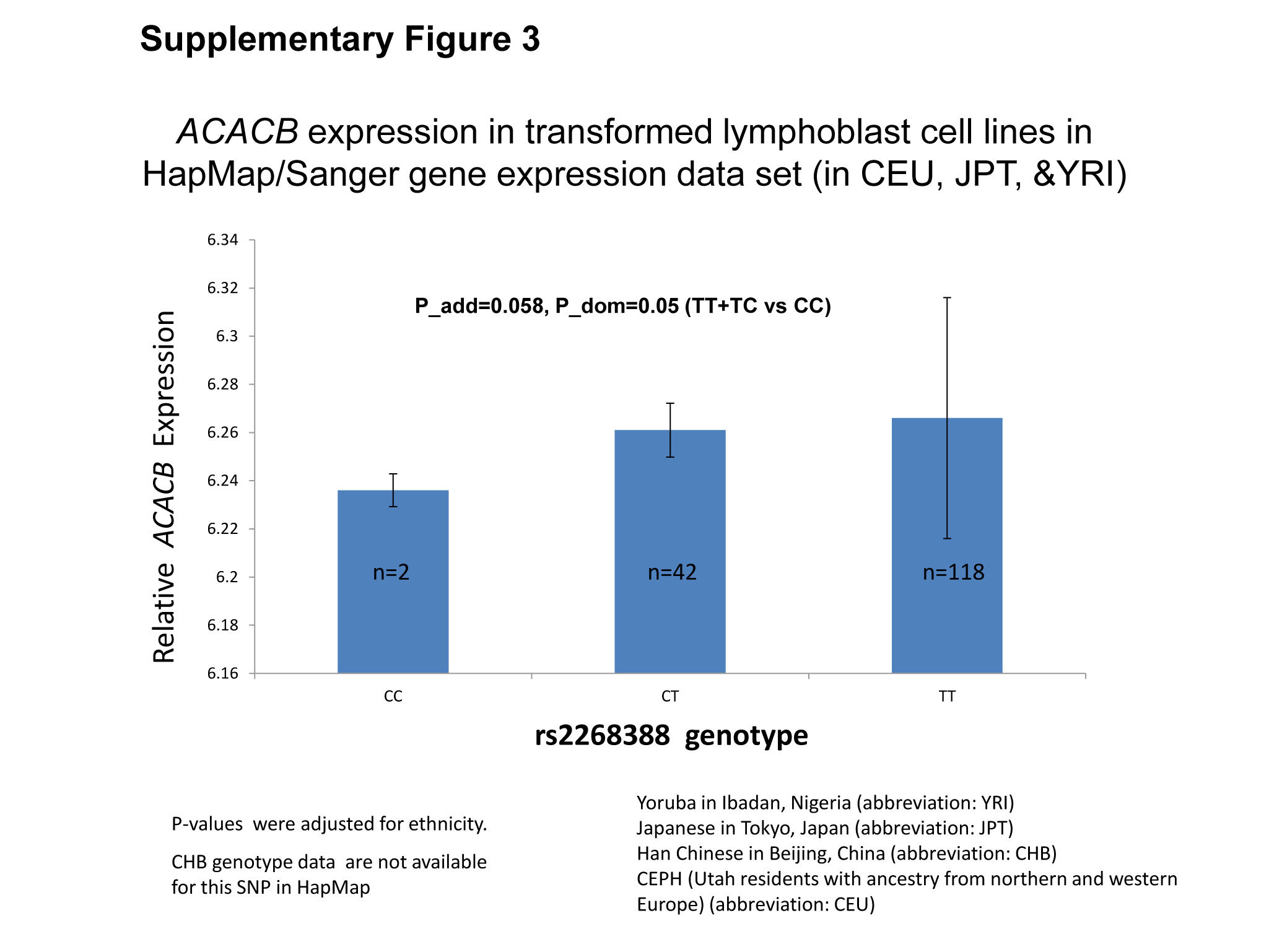

Supplement: Figure S3 — ACACB expression in transformed lymphoblast cell lines in HapMap/Sanger gene expression data set (in CEU, JPT, &YRI). (JPG) [file pone.0056193.s003.jpg]

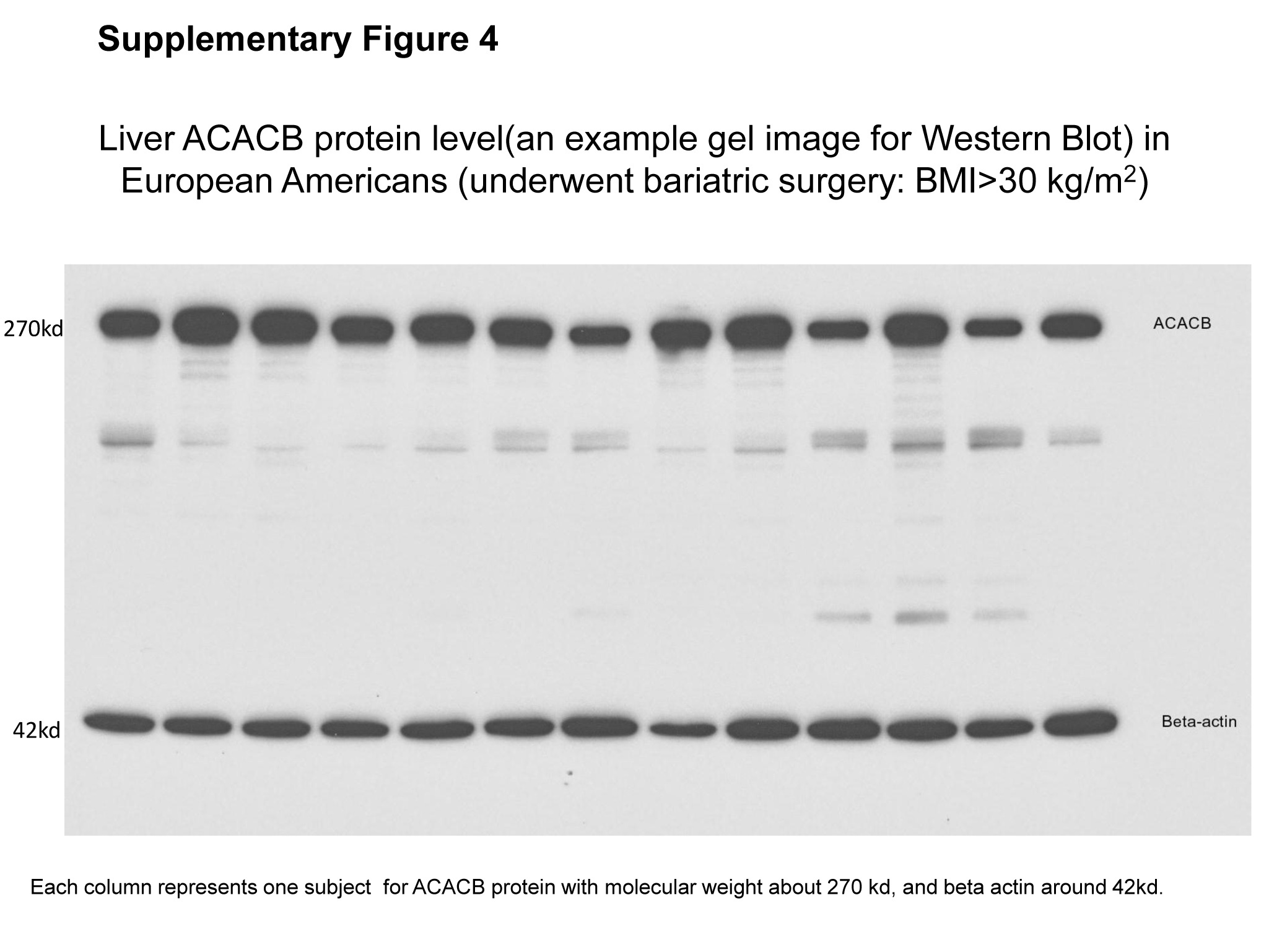

Supplement: Figure S4 — Liver ACACB protein level (an example gel image for Western Blot) in Caucasians (underwent bariatric surgery: BMI>30 kg/m2). (JPG) [file pone.0056193.s004.jpg]
