# Supplementary material for: An ACACB Variant Implicated in Diabetic Nephropathy Associates with Body Mass Index and Gene Expression in Obese Subjects
Source: PLoS One. 2013 Feb 27;8(2):e56193. doi: 10.1371/journal.pone.0056193 (PMC3584087; doi:10.1371/journal.pone.0056193)
Supplement: Table S1 — Association of rs2268388(C/T) with BMI in full-heritage Pima Indian men and women. (PDF) [file pone.0056193.s005.pdf]

Table S1 Association of rs2268388(C/T) with BMI in full-heritage Pima Indian men and women

Analysis	Gender		CC	CT	TT	p	p_interaction
Model 1	Men	n/N	198/1486	57/394	4/26		0.046
		Adjusted BMI z-score (mean±SE)	0.225±0.053	0.444±0.097	0.265±0.352	0.082	
		Raw BMI (mean±SD)	36.00±8.52	37.16±8.01	36.68±5.37		
	Women	n/N	272/2501	105/863	6/51		
		Adjusted BMI z-score (mean±SE)	0.224±0.049	0.347±0.072	-0.323±0.252	0.683	
		Raw BMI (mean±SD)	35.90±8.52	36.27±8.6	34.02±6.89		
Model 2	Men	n/N	995/5343	331/1765	27/129		0.435
		Adjusted BMI z-score (mean±SE)	-0.034±0.028	0.004±0.045	-0.004±0.149	0.478	
		Raw BMI (mean±SD)	34.20±7.89	34.38±7.76	33.75±8.3		
	Women	n/N	1372/9079	434/2800	38/269		
		Adjusted BMI z-score (mean±SE)	0.025±0.025	0.078±0.040	-0.076±0.126	0.546	
		Raw BMI (mean±SD)	34.20±7.90	34.71±8.35	33.25±7.03		

model 1: >=1 nondiabetic exam age <18 and subsequently developed DM; total n= 642; total exam N= 5321;

P values were adjusted for age, gender, sibship, and repeated exams (matrix: autoregressive; additive genetic model).

model 2: All individuals- all examinations ; total n=3197; total exam N= 19385;

P values were adjusted for age, gender, sibship, diabetic status, duration of diabetes, and repeated exams (matrix: autoregressive; additive genetic model).

T allele is defined as risk allele for higher BMI z-score.

n: number of individuals in each genotypic group. N: number of BMI exams.

P_ interaction refers to p value for genotype*gender interaction.
